# An experimental study of network effects on coordination in asymmetric games

**DOI:** 10.1038/s41598-019-43260-0

**Published:** 2019-05-02

**Authors:** Joris Broere, Vincent Buskens, Henk Stoof, Angel Sánchez

**Affiliations:** 1 Utrecht University, Centre for Complex Systems Studies, Utrecht, The Netherlands; 2 Utrecht University, Department of Sociology/ICS, Utrecht, The Netherlands; 3 Utrecht University, Institute for Theoretical Physics, Utrecht, The Netherlands; 4 Universidad Carlos III de Madrid, Grupo Interdisciplinar de Sistemas Complejos (GISC), Departamento de Matemáticas, Madrid, Spain; 5 Universidad de Zaragoza, Instituto de Biocomputación y Fisica de Sistemas Complejos (BIFI), Zaragoza, Spain; 6 Universidad Carlos III de Madrid-UV-UZ, Unidad Mixta Interdisciplinar de Comportamiento y Complejidad Social (UMICCS), Madrid, Spain; 7 Universidad Carlos III de Madrid, UC3M-BS Institute for Financial Big Data (IBiDat), Madrid, Spain

**Keywords:** Nonlinear phenomena, Complex networks

## Abstract

Network structure has often proven to be important in understanding the decision behavior of individuals or agents in different interdependent situations. Computational studies predict that network structure has a crucial influence on behavior in iterated 2 by 2 asymmetric ‘battle of the sexes’ games. We test such behavioral predictions in an experiment with 240 human subjects. We found that as expected the less ‘random’ the network structure, the better the experimental results are predictable by the computational models. In particular, there is an effect of network clustering on the heterogeneity of convergence behavior in the network. We also found that degree centrality and having an even degree are important predictors of the decision behavior of the subjects in the experiment. We thus find empirical validation of predictions made by computational models in a computerized experiment with human subjects.

## Introduction

Coordination problems are numerous in everyday life^1–3^. While avoiding collusion in traffic, meeting one another or making an exchange, coordination is a vital part of the success of the interaction. Coordination processes are therefore arguably fundamental to understanding the functioning of social, economic and biological systems. Game theoretical models are often used to model coordination problems with a strategic interdependence among actors. The success of this method has led to a wide literature ranging from ‘two by two’ games to complex spatial multi-agent (network) models, and from theoretical studies to experimental tests^4–8^. The coordination problem can be especially difficult when agents do not share the same preferences for different options. These situations are often formalized by asymmetric coordination games, such as the ‘battle of the sexes’. Computational models^9,10^ and other theoretical studies^11,12^ show that network structure is an important predictor of behavior in iterated asymmetric games. Although these models convincingly show that there are network effects on equilibrium behavior, no empirical studies have been conducted to corroborate these findings. Previous research on network effects on other types of games, such as the Prisoners Dilemma, show that the predictions made by computational models are not always evident when tested empirically^13^. It is therefore crucial to test the predictions and assumptions made by these models. In this paper we empirically test predictions made by computational and theoretical studies in an experimental study with human subjects^9–12^.

The problem with asymmetric coordination games is that it is difficult to make behavioral predictions about the outcome of the game. Table 1 illustrates a ‘battle of the sexes’ game in which the two pure-strategy Nash equilibria are: both players play *α* or both players choose *β*. However, the players differ in their preference for the equilibria. Therefore, the game consists of an element of coordination and an element of competition between the players. There is also a mixed Nash equilibrium, but this equilibrium is inefficient, because the expected payoff is lower for both players compared to any of the pure-strategy Nash equilibria. Miscoordination is the most frequent outcome when human subjects play these one-shot games in an experimental setting^4,6^. Both players often choose the behavior according to their own preferred equilibrium, resulting in the lowest payoff for both players. The players often converge to one of the two equilibria when the game is played repeatedly^14–16^. In rarer cases players manage to switch simultaneously between the two equilibria, thereby obtaining the best outcome for both players^14^.

**Table 1:** Example payoff table asymmetric ‘battle of the sexes’ game, where the first entry is for player 1 and the second entry for the player 2.

	Player 2	*α*	*β*
Player 1	*α*	2, 1	0, 0
	*β*	0, 0	1, 2

The situation can be made more complex by adding another player. This means there are three players who have to make a decision between *α* and *β*, but again they differ in their payoff for choosing *α* or *β*. Although the complexity of the situation is increased in terms of the number of players, it is easier to predict the outcome. The payoffs are balanced pairwise between the players, but the global situation is not, because there will always be a majority for one of the options. Intuitively one can already anticipate that when three people have to decide between two options, and two prefer option *β* and only one prefers *α*, the most likely outcome will be *β*. The three player situation is illustrated in Table 2, in which the payoffs are the sum of the pairwise interactions of Table 1. Again there are two pure-strategy Nash equilibria: all players play *α* or all players play *β*. However, in this case the equilibria are not equivalent and therefore not equally likely. The equilibrium ‘all players play *β*’ yields a higher payoff for the two column players. Also, when the column players both play *β*, their payoff will at least be two, independent of what player 1 does. When the column players both play *α*, their payoff will be two maximally. No such coordination possibility exists for player 1 and 2 or 1 and 3. Assuming full information for all players, choosing *β* is the best option for both column players. Because the row player is aware of this, the best reply would be to play *β* as well, making ‘all players play *β*’ the behavioral prediction in an empirical setting.

**Table 2:** Example payoff table of a 3 player asymmetric ‘battle of the sexes’ game. The first entry is for player 1, the second entry for player 2, and the third entry for player 3.

	Player 2	*α*		*β*	
	Player 3	*α*	*β*	*α*	*β*
Player 1	*α*	4, 2, 2	0, 0, 0	2, 1, 0	0, 2, 2
	*β*	0, 1, 1	1, 2, 0	1, 0, 2	2, 4, 4

**Figure 1:**
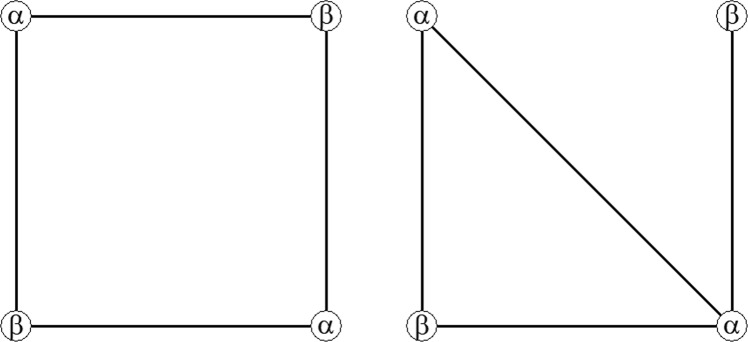
Four-player games, represented as a network; *α* and *β* denote the preferences of the players.

The situation changes again when players only interact with a subset of players in the game, instead of all other players. In Fig. 1 two situations with four players are illustrated. The nodes represent the players and the edges represent which nodes interact with each other. There are two nodes which prefer *α* and two nodes which prefer *β*. In the network on the left of Fig. 1 every node interacts only with nodes who have the opposite preference. Again the equilibria are: all players playing *α* or all players playing *β*. The situation is completely balanced, therefore it is again hard to predict the outcome of the game. The situation illustrated on the left of Fig. 1 is the same as the situation on the right of Fig. 1 with the exception that the edge from the top left node to the top right node is replaced with an edge from the top left node to the bottom right node. Although this is a minor change in the interaction structure, it is now easier to predict the outcome of this game. Both players with a preference for *β* always have to interact with players with a different preference. However, both players with a preference for *α* also interact with each other, making their pairwise interaction no longer an asymmetric battle of the sexes game, but a symmetric coordination game. Therefore, choosing *α* yields the highest payoff for the pairwise interaction between these two nodes. The node on the top right has no other choice than to coordinate on the same behavior as the node on the bottom right. The node on the bottom left can therefore infer that the other players will choose *α*, making it the best response to also choose *α*. So, ‘all players play *α*’ is the prediction in this situation.

In the 4-player situation one can still reason about what the predictions will be. In situations with more than 4 players it rapidly becomes harder to reason about the behavioral outcome of a game and the situation becomes yet more complicated when there are complex interaction structures added. In these types of situations, computational models are used to develop predictions.

There are several computational studies exploring the behavior of multiple agents with asymmetric ‘battle of the sexes’ type of dynamics. Several studies explore homogeneous spatial structures such as cellular automata^17–19^. An interesting finding is the ability of self organization in agreement clusters in cellular automata. Clusters of adjacent nodes can coordinate on one behavior while other parts of the cellular automata can coordinate on the other behavior. Hernández, Muñoz-Herrera and Sánchez introduce a theoretical model for exploring Nash equilibria of battle of the sexes games on Erdös-Rényi networks under the conditions of both complete and incomplete information^12^. They find a rich set of equilibria where both homogeneous and heterogeneous equilibrium behavior is possible when players have complete information. This set is reduced when players have incomplete information. In a subsequent study, the influence of group size and the strength of preferences on equilibrium behavior is studied^11^. The stronger the preferences, the harder it is to obtain homogeneous equilibrium behavior. Other related work studies computationally the behavior of dynamic networks in which actors can make or break their links^20^. This study identifies a set of networks that are stable, in the sense that actors no longer wish to break links or make new ones, often resulting in segregated networks. In addition, some studies consider the influence of heterogeneity in other games such as (weak) Prisoner’s Dilemma games^21–23^. An interesting result is that payoff heterogeneity is predicted to have a favorable effect on cooperation. Furthermore, there are some experimental studies with human subjects testing the effects of network structure on the number of rounds the participants need to reach coordination in both symmetric and asymmetric coordination games. Findings indicate that network structure influences the pace at which participants coordinate and network density can help the coordination process in asymmetry coordination games^24^. Other experimental studies show that with complete information on choices made by all actors in the entire network, participants are quicker in reaching coordination compared to more limited information sets^25^. However, these experimental studies do not study the effect of network structure on what the equilibrium behavior will be.

All this research, based mostly on computational models, yields specific predictions about global behavior on complex situations. However, obtaining those predictions often requires a lot of simplifying assumptions. The question then arises as to how well do these models predict actual behavior given the assumptions they make and the complexity of the situation? This is the main motivation for the experimental work we report in this paper.

## Theory

In this study we empirically test behavioral predictions from previous computational^9,10^ and theoretical studies^11,12^; specifically, we consider the influence of network structure on equilibrium behavior in iterated asymmetric ‘battle of the sexes’ games. We focus on the computational model described in Broere *et al*.^9^. In this study nodes play iterated 2 × 2 games with their neighbors. Each node gets a preference assigned before the first round of the game (say *α* or *β*), that determines whether the node is a row or a column player as shown in Table 2. Every round the nodes choose between *α* or *β* and the decision of the node is played against all its neighboring nodes. In the first round, the nodes play their preferred behavior with probability one. After every round, the nodes update their probability of playing *α* or *β* by means of reinforcement learning^26,27^. The probability of choosing either *α* or *β* is updated towards what would have been the best choice the previous round. Earlier research investigated the response behavior of human subjects in iterated ‘battle of the sexes’ games in two types of ring networks^28,29^. This study found that 96 percent of the decisions of the subjects followed a myopic best response pattern. We therefore choose a similar update rule. The update rule is more explicitly described in S1 of the Supplementary Materials.

The computational study compares the equilibrium behavior in three different types of networks^9^. The baseline model is the Erdös-Rényi (ER) random network. This type of network is usually defined as *G*(*N*, *p*_*er*_), where *N* is the number of nodes in the network and *p*_*er*_ the probability of drawing an edge between two arbitrary nodes. Thus, for every two nodes in the network the probability that an edge is present is the same. Depending on the choice of the parameters, this usually leads to a network with low clustering and relatively low variation in the degree distribution. The computational study predicts that equilibrium behavior is often homogeneous in these networks, in the sense that all nodes end up choosing the same behavior, all *α* or all *β*. However, it is hard to predict which equilibrium the nodes will end up playing, mostly because this is dependent on a complex combination of network characteristics. Both equilibria (all *α* or all *β*) are generally equally likely. This situation closely resembles the situations described in Table 1 and the left network of Fig. 1.

The second type of network studied in the computational study are clustered networks. The clustered networks are obtained by means of the Watts-Strogatz algorithm^30^. The algorithm starts with a lattice consisting of *N* nodes. Each node is connected with *n* neighboring nodes. The edges are rewired randomly with a constant probability for all edges. Using relatively low values of the rewiring probability, the characteristic of these networks are short average path lengths and high clustering, known as the ‘small world’ network characteristics. The computational study predicts that equilibrium behavior is homogeneous within the clusters, but heterogeneous between clusters, resulting in heterogeneous behavior in the overall network. Similar effects are shown in an experiment with different (learning) dynamics^31^. This result is pretty intuitive when taking a closer look at the definition of clustering. Clustering is often defined by the ratio of edges within a cluster and the edges outside the cluster^32,33^. The higher this average ratio, the higher the clustering of the network. In clustered networks coordinating on the same behavior within the cluster is simply more rewarding because there are on average more edges within the community than outside. This causes different communities to coordinate on different choices. A good predictor of the behavior within the cluster is the preference of high degree nodes. The higher the degree of a node, the higher the probability that the node converges to its preferred behavior.

The third type of network studied in the computational study are centralized networks. This type of network is constructed using the preferential attachment algorithm proposed by Barabasi and Albert^34^. This algorithm starts with one or more nodes and new nodes are added iteratively. In each iteration, the probability for a new node to connect to an existing node depends on the number of links the existing nodes already have; the more links a node already has, the higher the probability that the new node gets connected to it. This creates a ‘rich get richer’ effect. In the computational study, the centralized networks had low-clustering, a few central nodes with high degree centrality and a majority of peripheral nodes with low degree centrality. The equilibrium behavior in these networks is, just like random networks, often homogeneous in the sense that all nodes end up choosing the same option: all *α* or all *β*. However, which equilibrium the nodes converge to is easily predicted by the preference of the nodes with high degree centrality. In these networks the nodes with the highest degree centrality could dictate the equilibrium behavior of the overall network. This situation closely resembles the situation of the right network in Fig. 1.

Another effect which is present in all types of networks is related to an even degree centrality. Nodes with an even degree centrality can relatively easily coordinate on their preferred behavior compared to nodes with a similar but uneven degree centrality. This situation can be understood by reviewing the 3-player situation described in Table 2. When nodes have an even degree, the total number of relevant choices is uneven, including the node itself. So locally there is always a majority for one of the choices as described earlier in Table 2. Table 3 exemplifies the situation up to degree centrality eight. The percentage of neighboring nodes that have to choose the same behavior for a local majority is always 50 percent for nodes with an even degree centrality, while it requires more than 50 percent for nodes with an uneven degree centrality. This effect was also found in symmetric coordination games in a computational study by Buskens and Snijders^35^.

**Table 3:** Number of neighboring nodes required for a local majority given the degree of a node.

Degree centrality	1	2	3	4	5	6	7	8
Number of neighboring nodes needed	1	1	2	2	3	3	4	4
Percentage of neighboring nodes needed	100%	50%	67%	50%	60%	50%	57%	50%

Nodes with an even degree always need 50%, while an uneven degree requires more than 50% of its neighbors.

Summarizing, computational studies predict homogeneous equilibrium behavior in random networks, while the predicted probability is often close to 50 percent for either all *α* or all *β*. So, the information which can be derived from the network is limited. The predicted equilibrium behavior in centralized networks is also homogeneous, but the equilibrium behavior can be predicted by a few nodes with high degree centrality. In clustered networks degree centrality is also a good predictor of behavior, but behavior is restricted to the community the node belongs to. Finally, nodes with an even degree centrality more easily coordinate on their preferred behavior than nodes with an uneven degree centrality. Based on these computational predictions, we will empirically test the following hypotheses:

**Hypothesis 1:**

The correlation of (equilibrium) behavior between the computational model and the empirical results will be higher for centralized networks and clustered networks compared to random networks.

**Hypothesis 2:**

The equilibrium behavior is more heterogeneous in clustered networks compared to random and centralized networks.

**Hypothesis 3:**

Participants with high degree centrality will more often play their preferred behavior.

**Hypothesis 4:**

Nodes with an even degree centrality more often play their preferred behavior than nodes with uneven degree centrality in all networks.

## Methods

### Experimental setup

In order to test the four hypotheses we carried out a computerized laboratory experiment on networks of size 20. We used the Python based software platform oTree^36^. The experiments where conducted both at Universidad Carlos III de Madrid, Spain and Utrecht University, the Netherlands. In total 140 subjects participated in Madrid between April 9th and April 20th 2018. These participants were invited from the IBSEN volunteer pool^37^. In addition, 100 subjects participated between May 22nd and May 30th in the Experimental Laboratory for Sociology and Economics (ELSE), Utrecht. These participants were invited using the ORSEE recruitment system^38^. In total we conducted 12 sessions with 20 participants. Therefore N = 240 in total.

During the experiment, participants played an iterated asymmetric ‘battle of the sexes’ game against multiple other participants at the same time. Every participant interacted with a subset of other players. Interactions are mapped on a network in which the players are nodes and they play the game with the participants to whom they are connected. The payoff table is shown in Table 4. The payoffs are chosen such that the difference between coordinating on the preferred option and the not preferred option is a real difference, but small enough for participants to have incentive to deviate from their own preference. Participants can choose between ‘blue’ and ‘yellow’ and play the same choice against all participants they interact with.

**Table 4:** Payoff table for the experiment.

	Player 2	Blue	Yellow
Player 1	Blue	10, 8	0, 0
	Yellow	0, 0	8, 10

**Figure 2:**
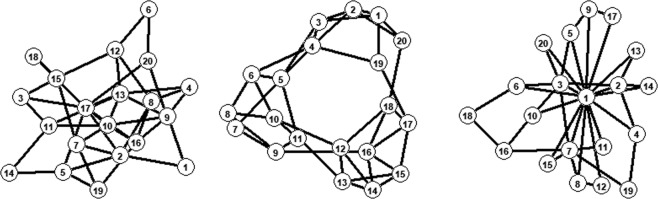
20-player games, represented as a network. Left the random network, in the middle the clustered network, on the right the centralized network.

In this study we used three different networks, each to represent the characteristics of random networks, clustered networks and centralized networks. The networks used in the experiments are represented in Fig. 2 and the adjacency matrices can be found in the Supplementary Materials S2, S3 and S4 of this paper. The network on the left in Fig. 2 has low clustering and low differentiation in degree centrality of the nodes. The network in the center of Fig. 2 has high clustering and low differentiation in degree centrality of the nodes. The network on the right in Fig. 2 has low clustering and a few nodes with high degree centrality. All networks have a network size of 20 nodes and a network density of 0.2. The network size is big enough that it contains non-trivial complexity and small enough to make it experimentally feasible. The computational study shows that the hypothesized outcomes can be expected for networks of size 20 and in a similar way for larger network sizes^9^.

Upon arrival participants were seated randomly in the laboratory. All subjects participated in games on all three networks. Before the first round of each network, participants were assigned their ‘type’ at random. The type determined whether they were the row or the column player in Table 4. The randomization was performed with the constraint of 10 row players and 10 column players to maximize the coordination problem. In every network, the participants played 20 rounds in which their type, location in the network, and the participants they interacted with are the same. The order in which participants were placed on each one of the networks was varied between sessions.

In each round participants had to decide between ‘blue’ and ‘yellow’. After all participants had made their decisions, they where all informed about their payoff in that round and how many of the participants he/she is connected with played ‘blue’ or ‘yellow’. The participant did not receive information about players in the network he or she was not connected with. Next, the participants were asked to make a new decision for the next round, continuing for 20 rounds. We informed the participants beforehand that they would play 20 rounds. We communicated the exact number of rounds because we did not expect any end game effects. Before the actual rounds were played the participants were asked to read the instructions of the game. After that, they were asked to answer questions about the instructions in order to test whether they understood the game. Before the participants played on the actual networks, three practice rounds on a random network were played.

The experiments lasted an average of 45 minutes. The payoff of each round was accumulated and then divided by the number of opponents (interaction partners in the network). For every 50 points earned, the participants received 1 euro. In addition, they received 5 euros show up fee. The maximum that could be obtained was 17 euros. The average payoff was around 14 euros, the lowest was 10.50 euros, the highest 16.50 euros. After the experiment, participants were asked to leave the room, after which they were invited one by one to collect their earnings in privacy. The experiments in Spain were in Spanish and the experiments in the Netherlands were in English. The instructions in both languages can be found in the Supplementary Materials S5 and S6 of this paper. The oTree code together with the obtained data and the R-scripts for the analyses can be found on the first authors github page: https://github.com/JJBroere/An-experimental-study-of-network-effects-on-coordination-in-asymmetric-games.

### Measures and analytical strategy

In order to test hypothesis 1, we have to calculate the correlation between the computational model and the empirical results. The correlations are computed with the same starting conditions; the same network and preferences of the actors in the same positions on the network. Given the same starting conditions, how often do the nodes in the computational model make the same choice (blue or yellow) as the people in the experiment? Thus, the correlation of the behavior between the computational graph *g*^*c*^ and the behavior of the empirical graph *g*^*e*^ for each graph is defined as:

1 $$r=cor({g}^{c},{g}^{e})=\frac{\sum _{i=1}^{N}\,\sum _{t=1}^{R}\,f({x}_{it}^{c},{x}_{it}^{e})}{N\times R},$$

where *x*_*it*_ is the behavior (‘blue’ or ‘yellow’) of node *i* at round *t*, *N* is the number of nodes and *R* the number of rounds. The function $f({x}_{it}^{c},{x}_{it}^{e})$ can have two values,

$$f({x}_{it}^{c},{x}_{it}^{e})=\{\begin{array}{cc}1 & {\rm{for}}\,{x}_{it}^{c}={x}_{it}^{e}\\ -1 & {\rm{for}}\,{x}_{it}^{c}\ne {x}_{it}^{e}\mathrm{.}\end{array}$$

The computational model is run with the same initial conditions (distribution of types) as the empirical result. Because the computational model has a stochastic update rule, we run the model 100 times and report the median correlation between the model and the empirical result. We chose to report the median, because in some cases the distribution can be very skewed. For the full specification of the computational model see S1 of the Supplementary Materials of this paper and Broere *et al*.^9^.

In order to test hypothesis 2 we resort to an analysis of variance (ANOVA) with post hoc tests on the heterogeneity of behavior in the networks in the last 5 rounds. We chose to look at the last 5 rounds instead of just the last round because random switching of behavior in the last rounds can influence the results. By taking the average of the last five rounds, the effects of switching or possible end game effects will be minimized in the analyses. We define heterogeneity of behavior as the variance of participants choosing ‘blue’ in the network:

2 $$h({p}_{blue})={\rm{var}}({p}_{blue})={p}_{blue}(1-{p}_{blue}),$$

where *p*_*blue*_ is the proportion of nodes playing ‘blue’ in the last 5 rounds of the game.

We test hypotheses 3 and 4 by means of a multilevel logistic regression for all subject decisions over all 20 rounds. Three levels are specified, taking into account the repeated measures of the participants and the correlation within networks. The dependent variable is 1 when the participant plays its preferred behavior and 0 otherwise. The predictor variables are the degree centrality of the participant in the network and the evenness of the degree.

## Results

In Table 5, the correlations between the empirical results and the computational model for each network are presented. We investigate how well the computational model predicts both the empirical behavior in all 20 rounds and the convergence behavior of the last five rounds. It is hard to define an objective convergence criterion, because participants sometimes switch behavior in an equilibrium state, presumably trying to persuade their neighbors to switch as well. We therefore apply a more subjective interpretation of convergence using as rule of thumb whether or not the switching of a player leads to switching behavior for other players as well. According to this interpretation, convergence is reached in the last five rounds in all sessions for the clustered network in Fig. 2, and for the network with the central node in Fig. 2. Not all sessions converged in the random network in Fig. 2. Six out of twelve networks did not fully converge in the last five rounds. This can occur because the switching of behavior was still effective, inducing neighbors switching behavior as well, while in the other two networks it rarely happened that switching behavior led to neighbors switching behavior as well. See S7, S8 and S9 of the Supplementary Materials of this paper for a visual example of the behavior of the computational model compared to the experimental results.

**Table 5:** Correlation between the computational model and the empirical results.

	All rounds		Last five rounds	
	Correlation (sd)	% correct (sd)	Correlation (sd)	% correct (sd)
Random	0.13 (0.09)	56 (4.61)	0.18 (0.16)	59 (8.29)
Clustered	0.44 (0.28)	72 (6.46)	0.51 (0.13)	76 (6.65)
Centralized	0.74 (0.17)	87 (8.74)	0.92 (0.25)	96 (12.48)
N	12	12	12	12

The correlation is defined in the text. The percentage correct is the percentage correctly predicted behavior of the experimental results by the computational model.

The correlation between the empirical results and the computational model is *r* = 0.13, *sd* = 0.09 for random networks over all 20 rounds and *r* = 0.18, *sd* = 0.16 for the last five rounds. The random network closely resembles the original two player game in Table 1 or the situation on the left in Fig. 1. In these situations different equilibria are equally likely. This is also reflected in the current results where the computational model poorly predicts the empirical behavior. The correlation of the clustered network is *r* = 0.44, *sd* = 0.28 and *r* = 0.51, *sd* = 0.13 for the last five rounds. This means that the model predicts 76 percent of the empirical observations correctly in the last five rounds. Predictions for these types of networks are significantly better compared to the random network. Finally, for the centralized network, the median correlation is *r* = 0.74, *sd* = 0.17 in all rounds and *r* = 0.92, *sd* = 0.25 for the last five rounds, meaning that towards the end behavior is predicted correct in almost all cases: in fact, out of 12 sessions the behavior was predicted wrongly only twice. In these cases the empirical behavior converged to the complete opposite direction from the predicted behavior, leading to a correlation of −0.98 and −1. This is reflected in the standard error. However, 10 out of 12 times the empirical results are correctly predicted by the computational model. Based on these results we found evidence in favor of hypothesis 1.

In order to test hypothesis 2, we examine heterogeneity of behavior in the last five rounds of the games for the different networks. The mean variance in the random networks is, *μ* = 0.116, *sd* = 0.099, although six out of twelve networks have not converged. The mean variance in the clustered networks is, *μ* = 0.183, *sd* = 0.085, corresponding to a situation in which clustered networks converged most of the time to a state where around half of the participants choose ‘blue’ and the other participants choose ‘yellow’. The mean variance in the networks with a central node is, *μ* = 0.024, *sd* = 0.030. This indicates that these networks converge to homogeneous behavior where all participants choose ‘blue’ or all participants choose ‘yellow’ every time. An ANOVA was conducted to compare the effect of the networks on the heterogeneity in the last five rounds. The ANOVA test yielded significant variation among conditions, *F*(2, 33) = 12.6, *p* < 0.001. A post hoc Tukey test further shows that the random networks and the clustered networks do not differ significantly at *p* = 0.101, although it should again be noted that the random network often did not converge. The clustered networks and the networks with a central node differ significantly at *p* < 0.001; the random network and the centralized network differ significantly at *p* = 0.019. Although the difference between the clustered network and the random network is not evident, we do believe that the random networks that did not converge yet would converge to the homogeneous state if more rounds where played. The random networks that did converge, all converged to a (mostly) homogeneous state. Furthermore, we found a very clear difference between the random network and the centralized network in terms of heterogeneity of behavior. We therefore conclude that we have empirical evidence in favor of hypothesis 2.

In Table 6 the results of the multilevel logistic regression analyses are presented. The dependent variable is a dichotomous variable indicating whether a participant chose the preferred behavior. The regression is conducted for all networks together, and with the three networks separately. In the model with all networks together there is a positive statistical significant effect of degree centrality on playing ones preferred behavior, *β* = 0.118, *sd* = 0.013, *p* < 0.001. The higher the degree centrality, the higher the probability that the participants will play their preferred behavior. There is also a positive statistically significant effect of an even degree centrality, *β* = 0.164, *sd* = 0.060, *p* = 0.006. If a participant has an even degree centrality, the probability that the participant will play his or her preferred behavior is higher as well. Looking at the random networks, there is no statistically significant effect of any of the predictors. In the clustered network there is a statistically significant effect of an even degree centrality, *β* = 1,033, *sd* = 0.476, *p* = 0.029. There is no statistical effect of degree centrality. In the centralized network we do find a statistical significant effect of degree centrality, *β* = 0.210, *sd* = 0.085, *p* = 0.013. However no statistically significant effect of having an even degree centrality. We found evidence in favor of hypothesis 3 and hypothesis 4 when combining all the data. However, the hypotheses could not be confirmed in the networks separately. This could be caused by a lack of statistical power and variation in the degree distribution within some types of networks.

**Table 6:** Multilevel logistic regression results, dependent variable is 1 if the participant chose his or her preferred behavior, 0 otherwise.

	All Networks	Random	Clustered	Centralized
Degree centrality	0.118*** (0.013)	0.185 (0.138)	0.280 (0.401)	0.210** (0.085)
Even degree	0.164*** (0.060)	−0.305 (0.473)	1.033** (0.476)	−0.400 (0.500)
Constant	0.228* (0.136)	1.171* (0.638)	−0.155 (1.638)	0.922* (0.493)
**Random effects**				
Subject	1.913 (1.383)	9.231 (3.038)	9.635 (3.104)	12.13 (3.483)
Network	0.017 (0.132)			
Observations	14,400	4,800	4,800	4,800
logLik	−7956.3	−1846.1	−1767.9	−1712.8
*Note:*	*p < 0.1; **p < 0.05; ***p < 0.01			

Three levels are specified, taking into account that repeated observations of behavior are nested within subjects, which are again nested within networks.

## Conclusion and Discussion

In this paper we empirically studied the effects of network structure on behavior in iterated ‘battle of the sexes’ games with human subjects. The hypotheses were derived from computational models from previous studies. The empirical results convincingly show that the computational models have empirical validity. The correlations between the computational model and the empirical behavior indicates that network structure governs the behavior to some extent. As expected, as the network structure becomes less ‘random’, the more accurate the computational model prediction of the behavior of the players. Furthermore, we found evidence that the major network effects found in the computational study have a clear counterpart on the behavior of the game when played with human subjects. Thus, in the clustered networks the equilibrium behavior was clearly more heterogeneous compared to the centralized networks. In random networks we did not find a significant difference with the clustered network. However, this could partially be explained because half of the sessions with the random networks did not converge after 20 rounds and more homogeneity could still be expected. We also found evidence that degree centrality and having an even degree plays a role in determining the behavior of the game.

Although the experiments were conducted in relatively small and simple networks, we do believe that the results are generalizable to bigger and more complex network settings. As both the computational and the empirical results show, most of the behavior is limited to the clusters of the network, and within each cluster our reasoning in terms of how majority influences decision presented above seems to apply quite well. When a larger network consists of multiple clusters, the same behavior can be expected within the clusters of the larger network. In larger networks with low clustering the same dynamics can be expected as in smaller networks, however the relative influence of a single node on the equilibrium behavior naturally becomes smaller as the number of nodes in the network becomes larger.

The small and limited number of networks studied is however a limitation of the current study. In this study we chose three fixed networks to represent networks used in the computational study; namely an Erdös-Rényi random network, a small world network and a preferential attachment network. There are several caveats to make about these choices. First of all, the networks belong to a class of network defined by their mathematical properties. We did not study any variation between networks within this class. The lack of variation within one network also makes it harder to detect the influence of different nodes. Secondly, the mathematical properties of the networks are often limiting properties as the number of nodes goes to infinity: although the effects are independent of size in the computational study, the networks in this study might in fact be too small to represent the limiting properties of the class of networks. However, studying larger and/or more different networks is complicated and expensive in an experimental context with human subjects, because this would require very large sample sizes and also a large amount of participant fee. The limiting factor is in the between network comparisons. In the current study it required 240 participants to be able to compare 12 networks per type.

All in all, our experiments show that the behavior of people trying to coordinate in a network can be understood reasonably well in terms of local considerations. In other words, subjects react to what they observe around them, the rest of the network does not seem to be relevant. This opens an interesting avenue of research as in other situations, different from the one considered here, global information may be available that changes how people behave. Indeed, it was observed that when the total number of people making one or the other choice is made available to the subjects, global coordination is reached much more easily in a population of moving subjects^39^. In the case of networks, knowing that there are very many players choosing the option one prefers may help to insist on making the decision that goes in one’s interest, in the hope that eventually one’s neighbors might conform to the global majority. This, along with trying to extend our experiments to larger networks and to more representatives of each class, would be an interesting contribution to establish the knowledge on coordination on networks on firm grounds.

## Supplementary information

Supplementary Materials
