# Supplementary material for: An experimental study of network effects on coordination in asymmetric games
Source: Sci Rep. 2019 May 2;9:6842. doi: 10.1038/s41598-019-43260-0 (PMC6497708; doi:10.1038/s41598-019-43260-0)
Supplement: Supplementary file 1 — Supplementary Materials [file 41598_2019_43260_MOESM1_ESM.pdf]

Supplementary materials: An experimental study of network effects on coordination in asymmetric games

Joris Broere [12], Vincent Buskens[12], Henk Stoof[13] & Angel Sánchez[456]

[1]Centre for Complex Systems Studies, Utrecht University, the Netherlands
[2]Department of Sociology/ICS, Utrecht University, the Netherlands
[3]Institute for Theoretical Physics, Utrecht University, the Netherlands
[4]Departamento de Matemáticas, Universidad Carlos III de Madrid, Spain
[5]Instituto de Biocomputación y Fisica de Sistemas Complejos (BIFI), Universidad de Zaragoza, Spain
[6]Unidad Mixta Interdisciplinar de Comportamiento y Complejidad Social (UMICCS), UC3m-UV-UZ, Spain
[7]UC3M-BS Institute for Financial Big Data (IFiBiD), Universidad Carlos III de Madrid, Spain

S1: Specification computational model

The 2x2 game can be mapped on a network by pairwise interactions between nodes who share an edge. In each round the nodes update their belief on what strategy yields the highest payoff, α or β, by means probabilistic dynamic in which the behavioral propensity changes towards the best response. Let $i = 1...N$ be the nodes in the population. Let $s \in \{\alpha, \beta\}$ be the strategy of node i, π_i the payoff of node i and π_i' the payoff when the alternative strategy would have been played. Then, the probability $p_{s,i}^t$ that a strategy s is played in round t, given the probability $p_{s,i}^{t-1}$ that a strategy s is played in round $t-1$ equals;

$$p_{s,i}^t = \begin{cases} p_{s,i}^{t-1} + 0.5 & \text{for } \pi_i^{t-1} \geq \pi_i^{\prime,t-1} \\ p_{s,i}^{t-1} - 0.5 & \text{for } \pi_i^{t-1} < \pi_i^{\prime,t-1}, \end{cases} \tag{1}$$

where $p_{s,i}^t$ is the probability that strategy s is played at some time t by node i (?, ?). So, at every time t each node updates the probability to play α or β towards a myopic best response reply strategy. If the best reply at time $t-1$ would have been α, the probability of playing α at time t increases compared to time $t-1$. If the best reply at time $t-1$ would have been β, the probability of playing α at time t decreases at time t (simultaneously the probability to play β increases). The probabilities are naturally bounded by the values 0 and 1. *Note: We used a step of 0.5 instead of 0.1, because we think this is more realistic for human subjects*

S2: Random network represented as adjacency matrix

Table 1: Adjacency matrix of the random network

0	1	0	0	0	0	0	0	1	0	0	0	0	0	0	0	0	0	0	0
1	0	0	0	1	0	1	1	0	0	0	0	0	0	0	0	1	0	0	0
0	0	0	0	0	0	0	0	0	0	1	0	0	0	1	0	1	0	0	0
0	0	0	0	0	0	0	1	1	0	0	0	1	0	0	0	0	0	0	0
0	1	0	0	0	0	0	0	0	0	0	0	0	1	0	0	1	0	1	0
0	0	0	0	0	0	0	0	0	0	0	1	0	0	0	0	0	0	0	1
0	1	0	0	0	0	0	0	0	1	0	0	0	0	1	0	0	0	1	0
0	1	0	1	0	0	0	0	0	0	0	1	0	0	0	1	0	0	0	0
1	0	0	1	0	0	0	0	0	1	0	0	1	0	0	1	0	0	0	1
0	0	0	0	0	0	1	0	1	0	1	0	1	0	0	1	1	0	0	0
0	0	1	0	0	0	0	0	0	1	0	0	1	1	0	0	0	0	0	0
0	0	0	0	0	1	0	1	0	0	0	0	1	0	1	0	0	0	0	0
0	0	0	1	0	0	0	0	1	1	1	1	0	0	0	0	1	0	0	0
0	0	0	0	1	0	0	0	0	0	1	0	0	0	0	0	0	0	0	0
0	0	1	0	0	0	1	0	0	0	0	1	0	0	0	0	0	1	0	0
0	0	0	0	0	0	0	1	1	1	0	0	0	0	0	0	1	0	1	0
0	1	1	0	1	0	0	0	0	1	0	0	1	0	0	1	0	1	0	1
0	0	0	0	0	0	0	0	0	0	0	0	0	0	1	0	1	0	0	0
0	0	0	0	1	0	1	0	0	0	0	0	0	0	0	1	0	0	0	0
0	0	0	0	0	1	0	0	1	0	0	0	0	0	0	0	1	0	0	0

S3: Clustered network represented as adjacency matrix

Table 2: Adjacency matrix of the clustered network

0	1	1	0	0	0	0	0	0	0	0	0	0	0	0	0	0	0	1	1
1	0	1	1	0	0	0	0	0	0	0	0	0	0	0	0	0	0	0	1
1	1	0	1	1	0	0	0	0	0	0	0	0	0	0	0	0	0	0	0
0	1	1	0	1	1	0	0	0	0	0	0	0	0	0	0	0	0	1	0
0	0	1	1	0	1	1	0	0	0	1	0	0	0	0	0	0	0	0	0
0	0	0	1	1	0	0	1	0	1	0	0	0	0	0	0	0	0	0	0
0	0	0	0	1	0	0	1	1	0	0	0	0	0	0	0	0	0	0	0
0	0	0	0	0	1	1	0	1	1	0	0	0	0	0	0	0	0	0	0
0	0	0	0	0	0	1	1	0	0	1	0	0	0	0	1	0	0	0	0
0	0	0	0	0	1	0	1	0	0	1	1	0	0	0	0	0	0	0	0
0	0	0	0	1	0	0	0	1	1	0	0	1	0	0	0	0	0	0	0
0	0	0	0	0	0	0	0	0	1	0	0	1	1	0	0	0	1	0	0
0	0	0	0	0	0	0	0	0	0	1	1	0	1	1	0	0	0	0	0
0	0	0	0	0	0	0	0	0	0	0	1	1	0	1	1	0	0	0	0
0	0	0	0	0	0	0	0	0	0	0	0	1	1	0	1	1	0	0	0
0	0	0	0	0	0	0	0	1	0	0	0	0	1	1	0	1	1	0	0
0	0	0	0	0	0	0	0	0	0	0	0	0	0	1	1	0	1	1	0
0	0	0	0	0	0	0	0	0	0	0	1	0	0	0	1	1	0	0	1
1	0	0	1	0	0	0	0	0	0	0	0	0	0	0	0	1	0	0	0
1	1	0	0	0	0	0	0	0	0	0	0	0	0	0	0	0	1	0	0

S4: Centralized network represented as adjacency matrix

Table 3: Adjacency matrix of the centralized network

0	1	1	1	1	1	1	1	1	1	1	1	1	1	1	0	1	0	0	1
1	0	1	1	0	0	0	0	0	0	0	0	1	1	0	0	0	0	0	0
1	1	0	0	1	1	1	0	0	1	0	0	0	0	0	0	0	0	0	1
1	1	0	0	0	0	0	0	0	0	0	0	0	0	0	0	0	0	1	0
1	0	1	0	0	0	0	0	1	0	0	0	0	0	0	0	0	0	0	0
1	0	1	0	0	0	0	0	0	0	0	0	0	0	0	0	0	1	0	0
1	0	1	0	0	0	0	1	0	0	1	0	0	0	1	1	0	0	1	0
1	0	0	0	0	0	1	0	0	0	0	1	0	0	0	0	0	0	0	0
1	0	0	0	1	0	0	0	0	0	0	0	0	0	0	0	1	0	0	0
1	0	1	0	0	0	0	0	0	0	0	0	0	0	0	1	0	0	0	0
1	0	0	0	0	0	1	0	0	0	0	0	0	0	0	0	0	0	0	0
1	0	0	0	0	0	0	1	0	0	0	0	0	0	0	0	0	0	0	0
1	1	0	0	0	0	0	0	0	0	0	0	0	0	0	0	0	0	0	0
1	1	0	0	0	0	0	0	0	0	0	0	0	0	0	0	0	0	0	0
1	0	0	0	0	0	1	0	0	0	0	0	0	0	0	0	0	0	0	0
0	0	0	0	0	0	1	0	0	1	0	0	0	0	0	0	0	1	0	0
1	0	0	0	0	0	0	0	1	0	0	0	0	0	0	0	0	0	0	0
0	0	0	0	0	1	0	0	0	0	0	0	0	0	0	1	0	0	0	0
0	0	0	1	0	0	1	0	0	0	0	0	0	0	0	0	0	0	0	0
1	0	1	0	0	0	0	0	0	0	0	0	0	0	0	0	0	0	0	0

S5: Instructions English

Instructions
These are the instructions for the experiment. These instructions are the same as on the screen. The instructions will remain available during the experiment. Please read the instructions carefully.

This experiment is divided into three main parts and an introduction part. In the introduction part three practice rounds will be played. During the three main parts of the experiment 20 rounds will be played. Before each part, you will be paired randomly and anonymously with 1 or more participants. The participants you are paired with will remain the same during the rounds of the different parts. So, in the main rounds the participants you are paired with will be the same for 20 rounds. After one part ends you will be randomly matched again. After the second random matching you probably play against other participants, but there is a chance they can also be the same.

Before each part you will be randomly designated a type. **Your type** can be Blue or Yellow. Prior to the start of the part you will learn which type you are. You will remain the same type during the rounds of the different parts. But before each part you will be randomly assigned a type again. *It will be clearly stated when a part ends and you will be assignment a new type and become randomly paired to new participants again.*

Each of you must independently and simultaneously make a decision between 'blue' and 'yellow'. **Your payoffs will be determined by the choices of the players you are paired with and the type you are.** Your **type**, Blue or Yellow, will indicate how many points you can earn with the **decision** 'blue' or 'yellow'. Below you find 2 tables, the table on the left for type Blue and the table on the right for type Yellow. In case you play against 1 other participant, when your type is Blue (left table), you choose 'blue' and the other participant also chooses 'blue', the payoff for that round is 10. When your type is Blue and you choose 'yellow' and the other participant also chooses 'yellow', the payoff for that round is 8. When you choose ‘blue' and the other participant chooses 'yellow' or the other way around, the payoff will be 0. When your type is Yellow (table on the right) you get the highest payoff of 10, when you and the other participant both choose yellow. 8 points when you both choose blue. And 0 point when you and the other participant don't choose the same colour.
Figure 1: Overview of possible profit points

Your type: Blue		**Your choice**	
		Blue	Yellow
Other participant’s choice	Blue	10	0
	Yellow	0	8

Your type: Yellow		**Your choice**	
		Blue	Yellow
Other participant’s choice	Blue	8	0
	Yellow	0	10

Note that your **type** will remain the same during the rounds of a part of the experiment, but you can change your **decision** every round. In this experiment you are randomly paired with 1 or more players who may or may not have the same type as you have. You will not be able to see which type the others are. You make one decision that holds for all participants you are paired with. The payoff of a round will be the average of the payoffs you get from the participants you are paired with. Thus, if you are paired with two other participants and you payoff with one would be 10 according to the table above and the payoff with the other would be 0, you earn 5 points in that round. At the end of the experiment, the points you earned will be paid out to you at an exchange rate of 50 points per euro.

S6: Instructions Spanish

Instrucciones

Estas son las instrucciones para el experimento. Estas instrucciones son las mismas que en la pantalla. Las instrucciones permanecerán disponibles durante el experimento. Por favor, lea atentamente las instrucciones.

Este experimento se divide en tres partes principales y una parte de introducción. En la parte de introducción se jugarán tres rondas de práctica. Durante las tres partes principales del experimento se jugarán 20 rondas. Antes de cada parte, se le emparejará de forma aleatoria y anónima con uno o más participantes. Los participantes con los que estás emparejado seguirán siendo los mismos durante las rondas de las diferentes partes. Por lo tanto, en las rondas principales, los participantes con los que estés emparejado serán los mismos durante 20 rondas. Después de que una parte termina usted será emparejado al azar otra vez. Después de la segunda coincidencia aleatoria, es probable que juegues contra otros participantes, pero existe la posibilidad de que también sean iguales.

Antes de cada parte se le designará un tipo al azar. Tu tipo puede ser azul o amarillo. Antes del comienzo de la parte usted aprenderá qué tipo usted es. Usted seguirá siendo del mismo tipo durante las rondas de las diferentes partes. Pero antes de cada parte se le asignará un tipo al azar de nuevo. Se indicará claramente cuando termine una parte y se le asignará un nuevo tipo y se le emparejará de nuevo al azar con nuevos participantes.

Cada uno de ustedes debe tomar una decisión de forma independiente y simultánea entre "azul" y "amarillo". Tus ganancias serán determinadas por las elecciones de los jugadores con los que estás emparejado y el tipo de jugador que eres. Tu tipo, Azul o Amarillo, te indicará cuántos puntos puedes ganar con la decisión "azul" o "amarillo". Debajo encontrará 2 tablas, la tabla de la izquierda para el tipo Azul y la tabla de la derecha para el tipo Amarillo. Durante los emparejamientos, cuando tu tipo es Azul (tabla de la izquierda), y eliges 'azul' y el otro participante también elige 'azul', el resultado de esa ronda es de 10 puntos, en cambio si ambos elegís 'amarillo' el resultado será de 8 puntos. De la misma forma, cuando tu tipo es Amarillo (tabla de la derecha) obtienes la máxima puntuación de 10 cuando ambos escogéis 'amarillo' y 8 cuando ambos elegís 'azul'. Si tú y el otro participante no escogéis el mismo color el resultado son 0 puntos.

Figura 1: Resumen de los posibles puntos de beneficio

Tu tipo: Azul		**Tu elección**	
		Azul	Amarillo
**Elección de otro participante**	Azul	10	0
	Amarillo	0	8

Tu tipo: Amarillo		**Tu elección**	
		Azul	Amarillo
**Elección de otro participante**	Azul	8	0
	Amarillo	0	10

Tenga en cuenta que su tipo seguirá siendo el mismo durante las rondas de una parte del experimento, pero puede cambiar su decisión en cada ronda. En este experimento usted es emparejado aleatoriamente con 1 o más jugadores que pueden o no tener el mismo tipo que usted tiene. No podrá ver de qué tipo son los otros. Tu elección en cada ronda se utilizará para resolver todos tus emparejamientos de esa ronda. El pago de cada ronda será el promedio de las recompensas obtenidas con todos tus emparejamientos. Por ejemplo, si usted está emparejado con otros dos participantes y en uno de los casos su recompensa es 10 puntos y en el otro es 0 su ganancia de esta ronda serian 5 puntos. Al final del experimento, los puntos ganados se le pagarán a un tipo de cambio de 50 puntos por euro.

S7: Example computational and experimental results random network

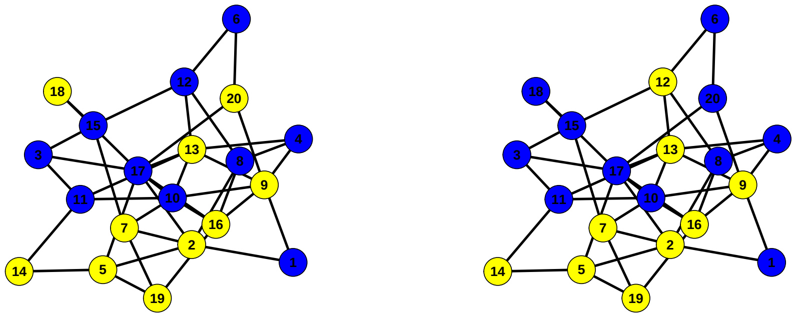

Figure 1: First round: Left computational model. Right: Experimental results

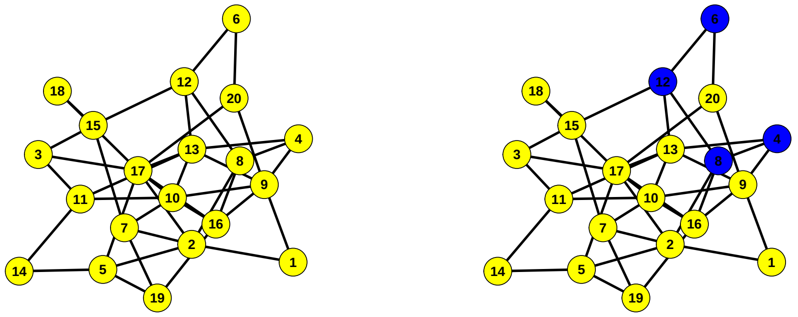

Figure 2: Last round: Left computational model. Right: Experimental results

S8: Example computational and experimental results small world network

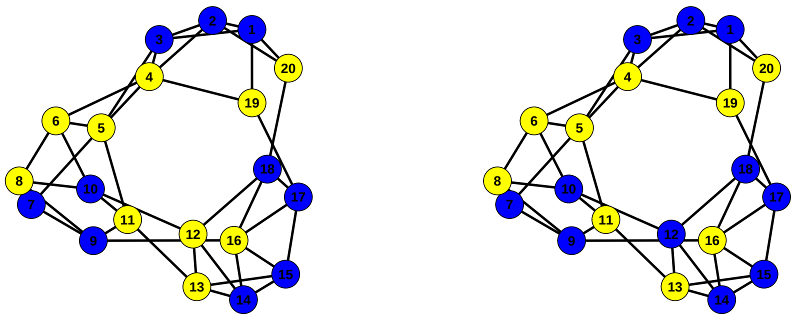

Figure 3: First round: Left computational model. Right: Experimental results

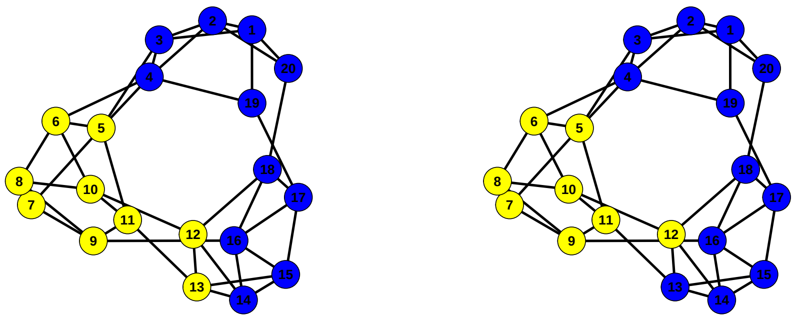

Figure 4: Last round: Left computational model. Right: Experimental results

S9: Example computational and experimental results Network with central node network

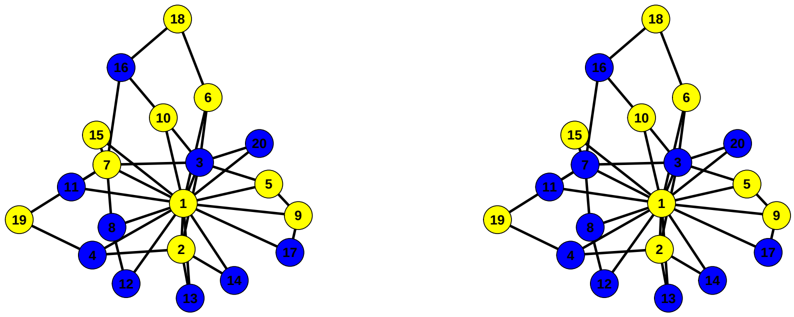

Figure 5: First round: Left computational model. Right: Experimental results

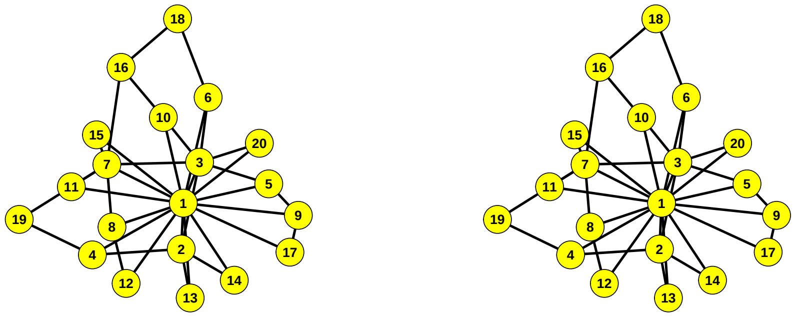

Figure 6: Last round: Left computational model. Right: Experimental results
